# Psychological Factors Affecting Risk Perception of COVID-19: Evidence from Peru and China

**DOI:** 10.3390/ijerph18126513

**Published:** 2021-06-17

**Authors:** Fredy S. Monge-Rodríguez, He Jiang, Liwei Zhang, Andy Alvarado-Yepez, Anahí Cardona-Rivero, Enma Huaman-Chulluncuy, Analy Torres-Mejía

**Affiliations:** 1 Department of Psychology, Universidad Nacional de San Antonio Abad del Cusco, Cusco 08002, Peru; 2 Department of Social Psychology, Nankai University, Tianjin 300350, China; jhpsy@mail.nankai.edu.cn; 3 School of Public Administration, Jilin University, Changchun 130012, China; zhanglw@jlu.edu.cn; 4 Instituto de Investigación Ambiente Comportamiento y Sociedad, Cusco 08002, Peru; psiko.andyjoss@gmail.com (A.A.-Y.); 164631@unsaac.edu.pe (E.H.-C.); 171143@unsaac.edu.pe (A.T.-M.); 5 Department of Pharmacy, Universidad Nacional de San Antonio Abad del Cusco, Cusco 08002, Peru; anahi.cardona@unsaac.edu.pe

**Keywords:** COVD-19, risk perception, anxiety, threat, confidence

## Abstract

COVID-19 has spread around the world, causing a global pandemic, and to date is impacting in various ways in both developed and developing countries. We know that the spread of this virus is through people’s behavior despite the perceived risks. Risk perception plays an important role in decision-making to prevent infection. Using data from the online survey of participants in Peru and China (*N* = 1594), data were collected between 8 July 31 and August 2020. We found that levels of risk perception are relatively moderate, but higher in Peru compared to China. In both countries, anxiety, threat perception, self-confidence, and sex were found to be significant predictors of risk perception; however, trust in the information received by government and experts was significant only in Peru, whereas self-confidence had a significant negative effect only for China. Risk communication should be implemented through information programs aimed at reducing anxiety and improving self-confidence, taking into consideration gender differences. In addition, the information generated by the government should be based on empirical sources. Finally, the implications for effective risk communication and its impacts on the health field are discussed.

## 1. Introduction

The outbreak of COVID-19 in early 2020 has had a global impact, and efforts to prevent further viral spread remain a priority. The high mortality rate has led to panic, stress, mental illness, and other burdens around the world. With the “structural violence” and vulnerability COVID-19 brings, many people are aware that they are at constant risk of infection [1]. Risk perception, or relying on individual intuitive risk judgments to assess various dangers in any given context [2], plays an important role in human self-protection, and social behavior [3]. Risk perception influences individuals’ decisions and behaviors. Many studies have demonstrated a link between risk perception and prevention of COVID-19 [4,5,6]. As the pandemic becomes more complex, there is a need for a better understanding of the public perception of risk [7]. 

This study follows the “holistic” approach proposed by van der Linden [8], which recommends including emotional, cognitive, social, cultural, and socio-demographic factors in the study of risk perception. Furthermore, it is necessary to contextualize the variables in a COVID-19 pandemic scenario [5]. In this sense, we assessed risk perception by considering the following psychological factors: anxiety, threat perception, trust in government information, self-confidence, and socio-demographic data such as sex, age, and nationality. On the other hand, this study selected data from China and Peru. The reasons are as follows: The first COVID-19 case appeared in Wuhan, China, and the Chinese government subsequently launched a strong response, demonstrating the value of dictatorship in containing viral spread [1]. At the time of this research investigation (July 2020), the virus had been basically controlled, and economic activities and public life had gradually recovered. Since Peru confirmed the first imported case on 6 March 2020, social distancing measures were immediately adopted. The total number of cases was still increasing rapidly at the time of the investigation of this study [9]. Therefore, in two countries at different stages of the epidemic, it is worth exploring whether there are differences in factors affecting people’s risk perception. Therefore, the present study has two goals. First, to assess which psychological factors have the best explanatory power on risk perception in the total sample. Second, to analyze which psychological factors best explain risk perception in each of the countries, Peru and China.

In this section, we detail the conceptual framework for each of the study variables.

### 1.1. Risk Perception and Anxiety

Evidence suggests that emotions hold an important role in risk perception. An experiment selecting 11 emotional indicators (such as worry, satisfaction) to verify the influence of emotions on risk perception of mobile telephones, genetically modified food, and terrorism respectively showed that all of these emotions are important factors in perceived risk. Negative emotions play the most important role [10]. Another similar study confirmed that emotions have a significant impact on everyday risk perception on more than 30 occasions [11]. In general, when experiencing negative emotions, people perceive more risks. Because a negative emotional state often interferes with information processing, leading to overestimation of potential future risks [12]. 

Anxiety is a common negative emotion in a major infectious disaster. There is a theory that risk and threat materials are more favored in the stages of cognitive processing for anxious individuals, and individuals with high anxiety had difficulty in attentional disengagement of negative stimuli such as risky materials [13]. Thus, anxious individuals also tend to experience higher risk perceptions. Direct evidence shows that there is a weak positive correlation between anxiety and risk perception [14], similar results were shown in COVID-19 research [15] Therefore, this study also hypothesizes that anxiety can positively predict risk perception.

### 1.2. Risk Perception and Perceived Threats

Risk is a quite broad concept in various fields. It is regarded as a threat to life or health in medical and hazard research or the probability that returns are not guaranteed in economic and commercial research. In the field of health risk perception, a common assumption is that fear is driven by the extent to which individuals regard the hazard as a serious and possible threat to health (perceived threat). Furthermore, other studies confirm that fear is a predictor of risk especially in the health field, linked to COVID-19 [16]. The majority of typical health behavior models emphasize the importance of perceived threats, such as Protection Motivation Theory (PMT) [17,18], which is a social cognition theory developed to understand how people respond to health threats. According to PMT, an individual’s intention of protective behavior is determined by threat and coping appraisal which are two parallel cognitive processes. In PMT, threat assessment emphasizes the severity of health threats, the susceptibility to threat events, and the performance of maladaptive behaviors. When individuals have a high-threat appraisal, they are more likely to engage in protective behaviors [19]. Additionally, examining the literature on terrorism risk perceptions, it is also found that risk perception is a multidimensional concept, usually measured from the dimensions of the possibility, severity, controllability, and unknown of the perceived threat [20]. In the field of health and safety, perceived threat is the core component of risk perception [20,21], that is, the higher the assessment of the severity of unknown threats, the stronger the risk perceived by individuals. The main risks of COVID-19 include risks to health and even life safety, so this study assumes that virus threat perception can actively predict risk perception.

### 1.3. Risk Perception and Trust in the Information of Government

The previous study identified the access to information as include mass media (such as television, newspapers), specialized media (such as government agency, experts), and interpersonal communication (such as friends and family) [22]. Some literature labeled specialized media as “formal information”, while information from interpersonal sources is regarded as “informal information” [23]. Trust in information can influence risk perception. Trust in informal information (TII) is approximated to relational trust which is based on judgments of the intentions of others, and trust in formal information approximates to calculative trust which is based on judgments of competence [24]. Research during the 2009 influenza A/H1N1 pandemic found that TII was statistically significantly positively associated with the risk perception of disease [25]. Similarly, a study on trust in messages and risk perception during avian influenza in Taiwan found that the higher the trust consumers have in messages about avian influenza, the lower their risk perceptions are [26]. Recent research also found that trust in official information sources can significantly positively predict confidence, and significantly negatively predict risk perception. Timeliness and transparency of information disclosure, trust in official and unofficial information sources, and confidence in pandemic prevention and control can explain 9% of the variation in risk perception [27]. Therefore, in this COVID-19 epidemic, we similarly put forward hypotheses: the higher the level of trust in the information of government, the lower the degree of risk perception of COVID-19.

### 1.4. Risk Perception and Self-Confidence

Confidence refers to the degree to which an individual feels capable of his own decisions and behaviors [28]. A Trust, Confidence and Cooperation (TCC) model for describing both trust and confidence in an entity responsible for the management of a particular hazard and risk perception is proposed. It acquiesces that there is a substitution that can be made between confidence and risk perception in a simplified TCC model. Confidence is the belief that certain events will occur as expected, which is another expression of perceived threat [29]. According to Mitchell [30], an increase in self-confidence represents a decrease in perceived risk. The main discussion on the relationship between confidence and risk perception is in consumer behavior. There is a correlation between specific self-confidence and strategies to reduce customers’ perceived risk [31]. Studies in the field of online transactions believed that people with a higher level of self-confidence will rarely perceive the loss associated with risk perception [32]. In neurobiology, it is believed that self-confidence has the function of mental strength and positive attitude and can be used as an intervention to reduce the psychological stress of patients. It has been theorized that self-confidence can help reduce the adverse experience of COVID-19 risk by controlling an acute immune response [33]. Although there is rare literature directly pointing out the relationship between self-confidence and risk perception of COVID-19, we can still assume, based on the above materials, that when an individual has a higher level of confidence, his perceived risk may be lower.

### 1.5. The Influence of Sex, Age, and Nationality on Risk Perception

Epidemic risk perceptions differ in demographic variables. Women seem to be more concerned about the risks related to the epidemic, environment, technology, and perceive higher threats [34,35,36]. In most environmental cases the risk perception levels of women are always higher than men [37]. Based on previous studies, sex differences in health and illness risk perception are relatively stable [38], and it was also demonstrated in many COVID-19 studies that women have a higher risk perception of COVID-19 [4,5].

The differences in factors such as age are still controversial. For nuclear facilities and other environmental factors, young people perceive a higher risk than older people [39,40]. However, there are also studies showing that age is positively associated with risk perception [41].

Many cross-cultural cognition studies have found that individuals in the same culture have similarities in risk perception, and there are group differences in risk perception between different cultures [42,43]. Cultural cognition theory emphasizes that the relationship between risk perception and cultural cognition is influenced by the traditional heuristic thinking process. There is an interaction between heuristic mechanisms and cultural values, which together affect individual risk perceptions [44].

Therefore, we also explore the influence of sex, age, and nationality on individual risk perception in this study.

## 2. Methods

### 2.1. Participants

The participants were recruited from China and Peru, with *N* = 502 and *N* = 1092, respectively. There were a total of *N* = 1594 participants. 

### 2.2. Procedures

Data were collected using online platforms throughout Peru and in 33 provinces of China, sampling was non-probabilistic for convenience. The virtual surveys were collected between 8 July and 31 August 2020. As of 29 July 2020, the official portal of the Peruvian state, reported 353,590 positive cases of COVID-19 being one of the countries with rapid advance of infections, causing 13,187 deaths; therefore, which, in addition to the slogans such as social distancing, isolation, and quarantine, the mandatory use of a face shield was implemented. In China, the number of infected to date was 83,600 and 4634 deaths; however, already had a high rate of recovery. The WHO website indicated that the inhabitants of China presented behaviors of staying more at home, washing their hands more frequently and wearing a mask when leaving to avoid contagion.

In Peru the platform used to collect data was Lime Survey, which is a free software application for conducting online surveys,. Regarding Peru 1552 questionnaires were collected. In order to guarantee the quality of the data, participants were excluded from the sample if they had these types of responses: incomplete answers people who had marked the same answers in more than two variables continuously (we know this way of answering as lazy answers, that is, without intention or answer variation), people who answered the first questions and the rest did not complete the survey. Therefore, 460 cases were excluded, leaving a data of 1092 valid subjects. Respect to China, a total of 563 questionnaires were distributed to the general public using the Wenjuanxing online survey, which is a common form of data collection in China. After excluding incomplete or invalid questionnaires, 502 valid responses were collected.

The data were processed by R-Studio, which is an integrated development environment for the R Programming Language, which is dedicated to statistical and graphical computing [45]. In terms of ethical procedures, the ethical principles of the Declaration of Helsinki were considered. All participants filled out the informed consent document, which guarantees the rights of the study participants. In addition, the study was reviewed by the Institutional Bioethics Committee of National University San Antonio Abad del Cusco registration number CBI-006-C2020-002.

### 2.3. Measurements

**Anxiety.** The GAD-7 consists of 7 questions scored on a scale ranging from 0 to 3 (e.g., “not being able to stop or control worrying”), with minimum and maximum possible scores being 0 and 21 respectively, and is used to assess general anxiety [46]. On the evaluation of internal consistency for the present study Cronbach’s Alpha was α = 0.93.

**COVID-19 Perceived Threat Questionnaire.** Composed of four questions, assessing the likelihood, and severity of a possible COVID-19 infection (e.g., “how serious do you think the new coronavirus infection would be if you contracted it?”), with minimum and maximum possible scores being 0 and 20 respectively, the alternatives are presented on a scale from 1 = very unlikely to 5 = very likely. On the evaluation of internal consistency for the present study Cronbach’s Alpha was α = 0.53 [47].

**Questionnaire on the extent of government information to the public.** Consists of two questions, assessing the degree of information received about the outbreak of the new coronavirus from the government [47] (e.g., “how often have you been confused or concerned about the reliability of the information you received from the government?”), with minimum and maximum possible scores being 0 and 10 respectively. The higher the score the items indicate more confusion and insufficient information on the part of the government.

**Self-confidence.** This aspect was assessed by a question “do I think I can take steps to protect myself against coronavirus?” to measure self-confidence in responding to the COVID-19, on a 5 point scale from 1 = strongly disagree to 5 = strongly agree [47].

**COVID-19 Risk Perception Scale.** The scale was assessed with 6 items to evaluate the perception that the new coronavirus COVID-19, represents in terms of risk [5] (e.g., “how likely do you think it is that you will be directly and personally affected by the following in the next 6 months?” contracting the coronavirus/COVID-19”), with minimum and maximum possible scores being 0 and 36 respectively. It has a scale from 1 = not likely to 7 = very likely, for this study this scale has an alpha of α = 0.72. The index comprises items that capture the perceived severity of COVID-19 pandemic participants, the perceived likelihood of contracting the virus themselves over the next 6 months, the perceived likelihood that their family and friends will contract the virus, and their level current concern about the virus [5]. All measurements can be found in the Supplementary material S1.

### 2.4. Statistic Analysis

A normality test was performed which confirmed that the data were non-parametric. Correlation analyses between variables were performed using Spearman’s Rho, the significance index was adjusted to 0.01 to be stricter. In addition, hierarchical multiple regression analyses were performed to see the explanatory power of the variables on COVID-19 risk perception. With respect to group comparisons, three-group scales were created (high, medium and low, the higher the score, the higher the perception of COVID-19 risk. The Kruskal–Wallis test was used for three groups and the Mann–Whitney U test for two groups of nonparametric data.

## 3. Results

### 3.1. Sample Characteristics

The age was grouped into three ranges: Young people aged 18 to 38 years (84.1%), adults aged 39 to 59 years (15%), and people over 60 years (0.9%), with a mean age of 30.8 years (SD = 106.01). In Peru, the sex was predominantly female with 56.1% (*N* = 613) and the remainder male with 43.9% (*N* = 479); the mean age of participants in Peru was 31.91. In China, the sex of the participants was also predominantly female with 58.6% (*N* = 294) and the rest male with 41.4% (*N* = 208); the average age of the participants in China was 28.53.

The groups related to risk perception differed significantly in terms of perceived threat levels (*X*^2^ Kruskal–Wallis (4) = 532.71, *p* < 0.001, ε^2^ˆordinal = 0.33), anxiety levels (*X*^2^ Kruskal–Wallis (3) = 109.53, *p* < 0.001, ε^2^ˆordinal = 0.07), also to the question “Is the information I have received from the government about the new coronavirus outbreak enough?” (*X*^2^ Kruskal–Wallis (4) = 69.82, *p* < 0.001, ε^2^ˆordinal = 0.04), with the question “how often have you felt confused or concerned about the reliability of the information you received from the government?” (*X*^2^ Kruskal–Wallis (4) = 162.85, *p* < 0.001, ε^2^ˆordinal = 0.10). They did not significantly differ to self-confidence “do I think I can take steps to protect myself against the coronavirus?” (*X*^2^ Kruskal–Wallis (4) = 5.42, *p* < 0.247, ε^2^ˆordinal = 0.00), neither with age (*X*^2^ Kruskal–Wallis (2) = 4.20, *p* = 0.123, ε^2^ˆordinal = 0.00). In general, it can be observed that the levels of risk perception are relatively moderate, but higher in Peru compared to China. We expose the sex and the country in the following section. Supplementary material S2.

### 3.2. Group Comparisons

In Table 1, the groups related to risk perception to COVID-19 differed significantly in terms of sex (log *W* (Wilcoxon) = 12.84, *p* ≤ 0.001, r = 0.15), country (log *W* (Wilcoxon) = 13.03, *p* ≤ 0.001, r = 0.54). The differences in the levels of the variables per country can be observed in the Supplementary material S3.

### 3.3. Correlations

In Table 2 we observe that people who have a higher perception of risk also have a higher perception of risk threats for COVID-19 r = 0.49 **. We also observe that anxiety has significant correlations with risk perception and perceived threats r = 0.31 ** and r = 0.20 **, respectively. Trust in government information was significantly correlated with perceived threat r = 0.12 ** and with anxiety r = 0.10**. In addition, trust in government information was significantly correlated with perceived threat r = 0.12 **, with trust in government information r = 0.17 ** and a negative correlation was observed with anxiety r = −0.08 **. Age was significantly correlated with perceived threat r = 0.08 **.

### 3.4. Regressions

In Table 3, we can see the results of a multiple OLS regression with risk perception as the dependent variable in the total sample of Peru and China (*N* = 1594). Starting from a baseline model, the influence of anxiety on risk perception is presented in model 1. The results show that the presence of anxiety is a significant predictor explaining 8% of the variance in risk perception (*F* (1, 1592) = 148, *p* < 0.001). In other words, the presence of anxiety is associated with an increased risk perception.

Model 2 tested whether perceived threats explain any additional variation in risk perception while controlling for anxiety. Inspection of the beta-standardized revealed significant effects (*F* (2, 1591) = 295.8, *p* < 0.001, adjusted *R^2^* = 0.27); indicating that perceived threats is a predictor associated with risk perception even when anxiety is controlled. Explaining an additional 19% of the variance risk of perception.

Model 3 explored the influence of trust regarding information received from the government about COVID-19, and perceived self-confidence to cope with the pandemic on risk perception, beyond the effect of anxiety and threat perception. It is observed that perceived self-confidence is a significant negative explanatory predictor (−0.08), explaining an additional 1% of the variance in risk perception (*F* (4, 1589) = 152.4, *p* < 0.001, adjusted *R^2^* = 0.28), trust about government information turned out not to be significant. Therefore, self-confidence is negatively associated with risk perception, which could indicate that the higher the self-confidence, the lower the risk perception.

Model 4 investigated the explanatory power of socio-demographic characteristics on risk perception, in addition to the degree of information and self-confidence, anxiety, and perceived threats. Country was found to be a significant predictor in the variance of risk perception, explaining 13% additional of variance, (*F* (7, 1586) = 153.7, *p* < 0.001, adjusted *R^2^* = 0.41). Furthermore, in model 4, it is observed that trust in government information becomes significant (in model 3 it does not), when adding the country variables, indicating that the country variable interacts with confidence in the information.

In the final (full) model 4, country, trust in government information, self-confidence, anxiety, and perceived threat for COVID-19 were identified as significant predictors, accounting for 41% of the total variance in risk perceptions for COVID-19, showing the greatest explanatory power. Age and sex were not significant.

Table 4 shows the results for Peru; the influence of anxiety following COVID-19 is presented in model 1. The results show that anxiety is a significant predictor explaining 8% of the variance in risk perception (*F* (1, 1090) = 93.74, *p* < 0.001, adjusted *R*^2^ = 0.08). In other words, understanding anxiety in the face of COVID-19 is associated with a higher risk perception.

Model 2 tested whether perceived threats explain any additional variation in risk perception, while controlling for COVID-19 anxiety. Inspection of the beta-standardized revealed significant effects (*F* (2, 1089) = 123, *p* < 0.001, adjusted *R^2^* = 0.18). Explaining an additional 10% of the variance of risk perception.

Model 3 explored the influence of trust regarding information received from the government about COVID-19, and perceived self-confidence to cope with the pandemic on risk perception, over and above the effect of anxiety and perceived threat. We find that trust of information is a significant explanatory predictor, explaining an additional 1% of the variance in risk perception (*F* (4, 1087) = 64.85, *p* < 0.01, adjusted *R^2^* = 0.19), but not self-confidence. Therefore, information is associated with risk perception, which could indicate that the more trust in the information from the government the more risk perception.

Model 4 investigated the explanatory power of socio-demographic characteristics on risk perception, while controlling for anxiety, perceived threats, degree of trust in government information, and self-confidence. Sex and age were not significant. This last full model explains 20% of the total variance, being the one with the highest explanatory power. This last full model accounts for 20% of the total variance, being the one with the highest explanatory power. This last complete model accounts for 20% of the total variance, being the one with the greatest explanatory power, with threat perception being the most significant.

In Table 5, of regressions for China, in model 1, show that anxiety is a significant predictor explaining 10% of the variance in risk perception (*F* (1, 500) = 56.72, *p* < 0.001, adjusted *R*^2^ = 0.10). In other words, the presence of anxiety is associated with an increased risk perception.

Model 2 tested whether perceived threat explains any additional variation in risk perception while controlling for the presence of COVID-19 anxiety. Inspection of the beta weights revealed significant effects (*F* (2, 499) = 63.09, *p* < 0.001, adjusted *R*^2^ = 0.20). Explaining an additional 10% of the variance of risk perception.

Model 3 explored the influence of trust regarding information received from the government about COVID-19, and perceived self-confidence to cope with the pandemic on risk perception, beyond the effect of anxiety and threat perception. Self-confidence is found to be a significant but negative explanatory predictor, explaining an additional 2% of the variance of risk perception (*F* (4, 497) = 36.39, *p* < 0.001, adjusted *R*^2^ = 0.22), but not trust in information. Therefore, self-confidence is negatively associated with risk perception, which could indicate that the higher the self-confidence, the lower the risk perception.

Model 4 investigated the explanatory power of socio-demographic characteristics on risk perception, in addition to anxiety, perceived threats, information received from the government about COVID-19, and self-confidence. Explaining an additional 1% of the variance risk perception (*F* (6, 495) = 24.89, *p* < 0.001, adjusted *R*^2^ = 0.23). Not significant predictors were found. Concluding that model 4, with an explanatory power of 23% of the variance in the perception of risk, better explains the perception of risk due to COVID-19 in China, with the perception of threat being the most significant.

## 4. Discussion

### 4.1. Predictors of Risk Perceptions

The regression model proved that, in all samples, anxiety is an important predictor of risk perception. The higher the anxiety level, the higher the perceived risk level. This is similar to the results of previous studies [14,15]. For example, a previous published report showed that ratings of negative events by participants with anxiety and depression were higher than people without anxiety and depression [49]. In the cognitive view of anxiety, there is a relationship between state anxiety (a transitory emotion trait caused by psychological arousal and consciously perceived feelings of apprehension, dread, and tension [50]) and the accessibility of information related to personal threat, while trait anxiety (individual’s predisposition to response [50]) is related to the degree or range of such information related to personal threat in memory [51,52]. According to previous findings, anxious individuals will overestimate the probability and severity of feared or negative events, and state anxiety would be associated with an increase in subjective risk in the judgment of negative events related to oneself [53,54]. This is also consistent with the trend found in this study. Multiple stressors can lead to increased levels of anxiety in an epidemic, and anxious persons will hold a higher risk perception of COVID-19. Excessive risk perception is likely to lead to a lot of irrational behaviors, so how to avoid excessive anxiety level has become a relatively important part.

The regression analysis also shows that threat perception is indeed an important positive predictor of risk perception. The higher the degree of perceived threat, the higher the level of risk perception. Similar to previous studies, perceived threat is an important component of perceived risk. In the social sciences, the most common way of analyzing risk is the realist approach, in which risk is seen as an objective threat or danger [55,56]. Therefore, risk perception can also be considered as the degree of perceived threat or danger. However, in this study, it can be seen that perceived threat is an important factor affecting risk perception, but not the whole factor. Evidence for this can also be found in other studies. For example, [57] measured the risk perception from three aspects: perception of health hazards, perception of hazards to the environment, and perception of general threats. In general, perceived threat is bound to be an important predictor of risk perception, but perceived threat can be divided into perceived threat to oneself, to friends and family, and threat in a more general sense. For example, studies have found that the general risk perception may be different from the perceived threat to oneself. Different types of threat perception are based on different foundations [58]. Moreover, it is also meaningful to explore the impact of different types of perceived threats on risk perception in future research.

The predictive effect of sex in the regression equation is also stable and significant in the Peruvian sample and the overall sample. The effects of sex on risk perception are consistent in previous studies, that is, risk perception level of female participants is always higher than that of male participants. For example, a study about risk perception and risk-taking behavior during adolescence found that compared with female participants, male participants perceived behaviors as less risky, were more prone to risky behavior, and were less sensitive to negative outcomes [59]. This suggests that the risk perception differences between men and women need to be further explored to find out what is the mechanism of this sex difference. In addition, it is also interesting to explore the interaction between sex and other predictors, which will be an important implication for future research.

The impact of information on risk perception is not consistent in different samples. An interesting finding is that trust in government information was significant in the total sample, and it is also significant for Peru, while for China it was not, with high levels of trust in government information being associated with low levels of risk perception, as confirmed by other studies [5]. This may be because the epidemic has been well prevented and controlled when the investigation was conducted in China (as of 31 July 2020, China had 276 new confirmed cases and one new death, according to the WHO.), and Chinese have passed the stage when there is a strong demand for information, so the importance of information trust has not been showed. Information overload, on the other hand, also explains this. Based on the theory of information processing, the filtering, control, maintenance and processing of information all need to use cognitive resources, and the finiteness of cognitive resources will directly determine the complexity and amount of information that an individual can process [60]. When information is overloaded, the continuous appearance of information will compete for limited processing resources. Too much information can affect an individual’s cognitive ability. So, it is very difficult to complete a series of cognitive operations, and human cognitive resources will be quickly exhausted, resulting in reduced decision-making performance [61]. This also serves as a reminder that in an emergency, as the stage of the event changes, more information is not always better. It should be targeted to provide a reliable amount of information. In addition, because Peru is at an important stage of the epidemic (according to the WHO, as of 31 July 2020, Peru has had 5678 new confirmed cases and 204 new deaths), the impact of information trust on risk perception reflects the real situation in the early stage of risk communication. That is to say, in the initial stage of the epidemic, formal information plays a very important role in risk perception. A study conducted in India during the early days of the COVID-19 found a high prevalence of misinformation, especially from social media, and it made people feel uncomfortable and distracts routine decision-making [62]. Thus, it is necessary to provide sufficient and reliable formal information in the epidemic to reduce the excessive risk perception of the public and avoid causing greater panic, especially in the initial stages of an epidemic, lacking accurate information. In addition, we have also found that the “country” may have an important influence on government information trust. Trust in government information has a significant effect on the overall sample in model 4. The reason is integrating “country” in model 4. The country is probably a moderator variable which increases the adjusted *R*^2^ to 0.41.

The overall sample and the Chinese sample show that the degree of self-confidence can significantly negatively predict the level of risk perception, while the Peru sample does not reach statistical significance, but it also shows a negative correlation. According to the previous theory, there are three main categories of risk neutralization strategies: scapegoating, self-confidence and risk comparisons. “Self-confidence” is what distinguishes individuals from “other people”, because they believe in their personal ability to avoid or control risky situations [63]. This is similar to the psychological construction of “unrealistic optimism”, which is described as a tendency to claim that they are less likely to be harmed than their peers [64,65]. That is to say, when an individual has a high level of confidence, his risk perception tends to be low. Our results do support this trend.

In addition, the conclusions of previous studies on age are not consistent. For example, a study investigating age changes in risk perception found that teenagers had lower risk perceptions of experimental, occasional, and regular involvement in 14 health-related activities (e.g., getting drunk) [37]. However, a research on the age difference between risk perception and unrealistic optimism found that teenagers minimized their involvement in health-threatening activities. Compared with their parents, teenagers are less optimistic about illness and avoidance of injury [66]. In this research, age has no significant predictive effect on risk perception.

Furthermore, there are some discoveries. We found that there were relatively few individuals with high levels of anxiety in this epidemic. This may be because the investigation in China was at a relatively controllable stage of the epidemic. In Peru, there are more people who perceive threats at medium and high levels, and this could be related to higher case numbers in Peru at that time. In addition, it has been observed that many media publish news about COVID-19, which have no scientific basis or is quite scientific and difficult to understand; therefore, people could be confused and present a greater perception of risk. However, most people have a high level of self-confidence and a medium level of information trust. In addition, Peru has a large distribution of people with a medium risk perception level, and China has a large distribution of people with a low risk perception level. Looking at the data for both countries in this study, it does not seem to show extreme levels of risk perception, which is an encouraging phenomenon. 

### 4.2. Implications for Effective Risk Communication

Risk communication can be seen as a base of accurate and scientific risk perception, which is about building trust by utilizing an interactive and ongoing communication process in which audience members are active participants [67,68]. Especially during the COVID-19 outbreak, ineffective risk communication will result in a wrong perception and health behavior, and further impede the effectiveness of risk management [69].

Our results reveal that people in Peru and China have similar risk perception characteristics, with anxiety and hazard perception proving to be the most significant predictors of risk perception in both countries. In addition, both countries have high levels of self-confidence. However, we found national differences, regarding the levels of risk perception, in the case of Peru 83% of the sample presents medium and high levels of risk, while in China 74% presents low levels of risk perception, these national differences could be explained by the fact that Chinese citizens have received information about the appearance and course of the new coronavirus since 2019 [70] on the other hand, in Peru, since the beginning of the state of emergency in March 2020, there was no accurate communication plan about the mechanisms of COVID-19 infection, which would explain a high perception of risk [71,72]. In fact, these characteristics actually have an impact on health behaviors, according to the serious situation of the COVID-19 outbreak. Based on these characteristics, the following implications for effective risk communication can be considered.

*Honest public policy for information disclosure.* Complete information disclosure about the risk can determine the level of emergency preparedness and response [73]. In view of Wuhan’s experience in China, insufficient information disclosure, to an extent, delayed the process of risk management, and resulted in more losses, including deaths and financial/economic losses [74]. Based on such situation, the political decision-maker should be responsible for information transparency to guarantee the public’s health and safety. By swift information disclosure, the public can conduct timely protection method and realize the severe situation of the public health emergency. Additionally, political decision-makers should use the evidence-based decision-making to cope with the professional issue in the public health emergency, which means that follow the epidemiological investigation and experts’ recommendations to make effective public policy to tackle the risk [75]. Briefly, earlier governmental intervention is related to less losses, and transparent information is associated with effective emergency preparedness [76].

*Using plain language to deliver professional public health knowledge.* Public health issues always be professional. Especially some medical terms are isolated from people’s knowledge domains. Due to diverse health literacy amongst people, different individuals have divergent capacities to understand and cope with health information [75,77]. Therefore, effective risk communication has to assess the different levels of perception among different audiences, and to use plain and understandable expressions to transfer the professional expressions. A vital principle is that “never assume technical knowledge about the issue unless the audience is clearly from a technical community.” [78]. Additionally, vivid or graphic metaphors for exemplification can illustrate the epidemiological characteristics in a direct, simple, and visual way, which can match people’s various health literacy [73,79]. The main objective of such method is to make the public know the real situation of the pandemic rather than standing traditional cognition.

*Applying for credible sources for establishing people’s trust in risk information.* Credible sources of health information is a main element of effective risk communication, and some research shows clearly that there is a positive correlation between low perceived risk and public trust, and vice versa [78]. Following China’s experience, credible expert’s statements had a quite significant impact on people’s perception and health behavior [73]. Therefore, a credible source of health information will change people’s misperception of public health risk and its consequences, and can help people choose scientific health behavior. For instance, expert opinion or commonly used social media as sources of information could disseminate protective methods and dynamic risk information. In addition, complete information transparency and open communication are the ways to gain or sustain trustworthiness. Therefore, accessibility and openness can enhance the public’s perception that they are fully informed about risk and that they are partners in sharing the risk, which will lead them to conduct accurate health behavior [73,78,80].

*Timely and often risk communication for combating health misinformation.* Risk communication ought to begin as soon as a risk has been identified or even before a risk has occurred (if it is possible to identify beforehand). and continue as new information is available [81]. Experiences in different countries have shown the passive consequences of delayed risk communication. Meanwhile, the COVID-19 outbreak also led to the spread of health misinformation that the World Health Organization (WHO) has called “infodemic” [82,83]. Such health misinformation misled people’s perception and even resulted in irrational behavior, such as stockpiling [84]. Therefore, the effective method for combating health misinformation is to communicate risk timely and often, which can eliminate the uncertainty and ambiguity of the risk [85]. Risk communication is a dynamic process. Effective communication is dependent upon continuous update of disease information, which enables the public to grasp timely accurate information in case the misinformation misleads people’s perception in advance.

*Limitations.* Participants are recruited through online questionnaire collection. The age distribution is relatively uneven and the representation is limited. Although the statistical method has been revised, future research still needs a more optimized method of participant recruitment. Finally, although the aim of the study was to identify the influence of variables such as anxiety, threats, information, confidence and socio-demographic factors on risk perceptions, future research should consider cultural elements that can more comprehensively explain risk perception in the context of a COVID-19 pandemic. In addition, the main limitation of this study is the use of convenience sampling for data collection. The unrandomized sample limits the generality of the research conclusions. Although a large size of the sample can make up for this shortcoming to some extent, the generality of the conclusion still needs to be treated with caution. This study explores the direct impact of the main factors that affect risk perception. However, the interaction between variables has not been further explored, and the reason why factors such as sex are not significant after adding other variables may be because other factors have played a moderating role. These more complex mechanisms of action need to be further explored in future research.

## 5. Conclusions

The present study presented the psychological predictors of risk perception in the context of the COVID-19 epidemic outbreak in samples from Peru and China. The results show that anxiety and threat perception are two stable influencing factors, and both are important predictors of COVID-19 risk perception, whether in China, Peru, or the overall sample. The results also confirm that self-confidence is also another influential factor in China, while trust in information is more important than self-confidence in Peru. Risk communication must have a commitment to developing an honest public policy for information disclosure, based on research results and expert opinion. In addition, the use of plain language and timely risk communication will ensure the health and safety of the public. From these experiences, we can learn that in pandemic situations, having a rapid response system on risk communication could help us to better manage the situation and consequently reduce the negative impacts.

*Recommendations for public health systems.* Primary health care systems and health promotion programs should, in the short term, develop campaigns aimed at reducing anxiety levels in populations by providing clear information to help citizens make the right decisions in the face of the COVID-19 pandemic. In addition, these messages should be differentiated for men and women, due to differences in risk perception. In the long term, the government should design a risk communication plan based on empirical information, and disseminate it through the mass media, which will allow people to develop greater confidence in health policies and also increase their self-confidence, all of which will help to reduce losses as a consequence of the COVID-19 pandemic.

## Figures and Tables

**Table 1:** Group comparisons of sex and country with risk perception.

Variable	Sample	Low	Medium	High	*W*	*p*
	Total	(*N* = 558)	(*N* = 776)	(*N* = 260)		
	(*N* = 1594)					
	*n* (%)	*n* (%)	*n* (%)	*n* (%)		
Sex					12.84	0.001 ***
Female	821 (51.5)	229 (27.9)	435 (53)	157 (19.1)		
Male	773 (48.5)	329 (42.6)	341 (44.1)	103 (13.3)		
Country					13.03	0.001 ***
Peru	1092 (68.6)	185 (16.9)	675 (61.8)	232 (21.2)		
China	502 (31.4)	373 (74.3)	101 (20.1)	28 (5.6)		

Note: *W* test (U Mann–Whitney) was used. *** *p* < 0.001.

**Table 2:** Correlations.

Variable	*M*	*SD*	1	2	3	4	5
1. Risk Perception	19.59	6.78					
2. Perceived Threat	12.96	2.85	0.49 **				
3. Anxiety	4.64	4.35	0.31 **	0.20 *			
4. Trust in government information	6.20	1.27	0.05 *	0.12 **	0.10 **		
5. Self-confidence	3.97	0.89	−0.02 (n.s.)	0.12 **	−0.08 **	0.17 ***	
6. Age	30.84	106.01	0.05 (n.s.)	0.08 **	0.03 (n.s.)	0.03 (n.s.)	0.01 (n.s.)

Note. *M* and *SD* are used to represent the mean and standard deviation, respectively. The values in brackets indicate the 95% confidence interval for each correlation. The confidence interval is a plausible range of population correlations that could have caused the sample correlation [48]: * *p* < 0.05, ** *p* < 0.01, *** *p* < 0.001, n.s. = not significant.

**Table 3:** Regressions PeruChina.

Independent Variables	Model 1 (β Stand.)	Model 2 (β Stand.)	Model 3 (β Stand.)	Model 4 (β Stand.)
Anxiety	0.30 ***	0.21 ***	0.21 ***	0.20 ***
Perceived Threats		0.44 ***	0.45 ***	0.30 ***
Trust in government information			−0.01 (n.s.)	0.05 *
Self-confidence			−0.08 ***	−0.06 **
Sex				−0.04 (n.s.)
Age				−0.02 (n.s.)
Country				−0.38 ***
*F*	148 ***	295.8 ***	152.4 ***	153.7 ***
Adjusted *R^2^*	-	0.27	0.28	0.41
*N*	1594	1594	1594	1594

Note: The dependent variable is the risk perception (index). It is also observed that the Country shows the highest predictive index of risk perception, with β = −0.38, then perceived threat with β = 0.30. The entries are standardized beta coefficients; * *p* < 0.05, ** *p* < 0.01, *** *p* < 0.001, n.s. = no significant.

**Table 4:** Regressions Peru.

Independent Variables	Model 1 (β)	Model 2 (β)	Model 3 (β)	Model 4 (β)
Anxiety	0.28 ***	0.21 ***	0.21 ***	0.20 ***
Perceived Threats		0.33 ***	0.31 ***	0.31 ***
Trust in government information			0.10 **	0.10 **
Self-confidence			−0.02 (n.s.)	−0.02 (n.s.)
Sex				−0.06 *
Age				−0.03 (n.s.)
*F*	93.74 ***	123 ***	64.85 ***	44.25 ***
Adjusted *R^2^*		0.18	0.19	0.20
*N*	1092	1092	1092	1092

Note: The dependent variable is the perception of risk (index). The entries are standardized beta coefficients; * *p* < 0.05, ** *p* < 0.01, *** *p* < 0.001 (n.s. = no significant).

**Table 5:** Regressions China.

Independent Variables	Model 1 (β)	Model 2 (β)	Model 3 (β)	Model 4 (β)
Anxiety	0.32 ***	0.30 ***	0.28 ***	0.28 ***
Perceived Threats		0.32 ***	0.32 ***	0.32 ***
Trust in government information			−0.002 (n.s.)	−0.001 (n.s.)
Self-confidence			−0.16 ***	−0.16 ***
Sex				−0.01 (n.s.)
Age				(n.s.)
*F*	56.72 ***	63.09 ***	36.39 ***	24.89 ***
Adjusted *R^2^*		0.20	0.22	0.23
*N*	502	502	502	502

Note: The dependent variable is the perception of risk (index). It is also observed that the perceived threats of COVID-19 show the highest predictive index of risk perception, with β = 0.32. The entries are standardized beta coefficients; *** *p* < 0.001 (n.s. = no significant).

## Data Availability

The data presented in this study are openly available in OSF at https://osf.io/bpmgh/ (accessed on 15 June 2021).

## Supplementary Materials

The following are available online at https://www.mdpi.com/article/10.3390/ijerph18126513/s1, Supplementary material S1: Survey example. Perceptions and Behaviors in the face of the New Coronavirus COVID-19. Supplementary material S2: Comparisons of risk perception with age, anxiety, perceived threats, questions 1 and 2 of degree of information, self-confidence, sex, and country. Supplementary material S3: Differences in the levels of the variables by country.
