# Supplementary material for: Psychological Factors Affecting Risk Perception of COVID-19: Evidence from Peru and China
Source: Int J Environ Res Public Health. 2021 Jun 17;18(12):6513. doi: 10.3390/ijerph18126513 (PMC8296494; doi:10.3390/ijerph18126513)
Supplement: Supplementary file 1 [file ijerph-18-06513-s001.zip › S1 measurement.pdf]

Supplementary material S1

Perceptions and Behaviors in the face of the New Coronavirus COVID-19

The new coronavirus (COVID-19) whose first outbreak was in the country of China, has caused several effects at all levels of development around the world, we want to see some effects that it has had on our population.

Welcome to our COVID-19 survey!

With your participation you will be supporting an investigation carried out by the Center for Research, Environment, Behavior and Society (CIACOMS) of the National University of San Antonio Abad del Cusco. With this research we seek to study the perceptions and behaviors related to COVID-19.

BEGINNING

The survey lasts approximately 25 to 30 minutes and your participation is completely voluntary. Your information will be kept anonymous and encrypted (that is, all the data that could identify you will be replaced by codes). The results of this research will be published anonymously in scientific reports and journals. At no time will information be displayed that allows the identification of the people who participate in this survey. Rather, the results represent general trends in the population.

Please note that our goal is not to evaluate or persuade you; we just want to know your opinion. You can freely answer what you think and best represents your point of view. You may also withdraw from the study if you deem it appropriate without causing any harm to you.

If you wish to have access to the first results obtained in this research, indicate it, also, at the end of the survey. The email addresses that are collected in this investigation will be separated from the data before analysis, will be kept confidential and will not be released to third parties.

If you have questions or need more information, feel free to contact us via email at fredy.monge@unsaac.edu.pe.

Thank you very much for your help!

DECLARATION AND / OR CONSENT

I voluntarily agree to participate in this study, I understand the activities in which I will participate if I decide to enter the study, I also understand that I can decide not to participate and that I can withdraw from the study at any time.

Please confirm:

*

Select one of the following options

Please select only one of the following options:

• I have read and understood the previous text and I agree to participate in this survey.

• I do not wish (for the moment) to participate in this survey.

PERSONAL INFORMATION

HERE WE REQUEST SOME PARTICIPANT DATA

Sex *

Please select only one of the following options:

- Feminine
- Male

Age *

Please write your answer here: ----------

ANXIETY: GAD-7	Not, at all	Several days	More than half the days	Nearly every day
	0	1	2	3
Feeling nervous, anxious or on edge				
Not being able to stop or control worrying				
Worrying too much about different things				
Trouble relaxing				
Being so restless that it is hard to sit still				
Becoming easily annoyed or irritable				
Feeling afraid as if something awful might happen				

Perceived threats to covid-19					
	very unlikely	2	3	4	very likely
	1	2	3	4	5
How likely do you think you will get the new coronavirus infection in the next month?					
	very mild	2	3	4	very serious
	1	2	3	4	5
How serious do you think the new coronavirus infection would be if you contracted it?					
	much lower	lower	similar	higher	much higher
	1	2	3	4	5
How transmissible is the new coronavirus (covid-19) compared to SARS (outbreak in 2002)?					

What is the damage to the body of the new coronavirus (covid-19) compared to SARS (outbreak in 2002) how is it?	

Trust in government information

	strongly disagree	disagree	Neither agree nor disagree	agree	strongly agree
	1	2	3	4	5
Is the information I have received from the government about the outbreak of the new coronavirus sufficient?					Reverse code
	never	rarely	sometimes	usually	always
	1	2	3	4	5
How often have you been confused or concerned about the reliability of the information you received from the government?					

Self -Confidence

	strongly disagree	disagree	Neither agree nor disagree	agree	strongly agree
	1	2	3	4	5
Do I think I can take steps to protect myself against the coronavirus?					

Risk perception

		Not at all worried	2	3	4	5	6	Very Worried
		1	2	3	4	5	6	7
1	How worried are you personally about the following issues at present? - Coronavirus/COVID-19							
		Not at all likely	2	3	4	5	6	Very Likely
		1	2	3	4	5	6	7

2	How likely do you think it is that you will be directly and personally affected by the following in the next 6 months? - Catching the coronavirus/COVID-19						
3	How likely do you think it is that your friends and family in the country you are currently living in will be directly affected by the following in the next 6 months? - Catching the coronavirus/COVID-19						
		Strongly disagree	2	3	4	Strongly agree	
		1	2	3	4	5	
4	How much do you agree or disagree with the following statements? - The coronavirus/COVID-19 will NOT affect very many people in the country I'm currently living in						Reverse coded
5	How much do you agree or disagree with the following statements? - I will probably get sick with the coronavirus/COVID-19						
6	How much do you agree or disagree with the following statements? - Getting sick with the coronavirus/COVID-19 can be serious						

Español

Percepciones y Conductas ante el Nuevo Coronavirus COVID-19

El nuevo coronavirus (COVID-19) cuyo primer brote fue en el país de China, ha ocasionado varios efectos en todos los niveles de desarrollo en todo el mundo, queremos ver algunos efectos que ha tenido en nuestra población.

¡Bienvenido/a nuestra encuesta sobre COVID-19!

Con su participación estará apoyando a una investigación realizada por el Centro de Investigación, Ambiente Comportamiento y Sociedad (CIACOMS) de la Universidad Nacional de San Antonio Abad del Cusco. Con esta investigación buscamos estudiar las percepciones y comportamientos relacionados al COVID-19.

INICIO

La encuesta dura aproximadamente 25 a 30 minutos y su participación es totalmente voluntaria. Su información será guardada de manera anónima y codificada (es decir, que todos los datos que podrían identificarle serán sustituidos por códigos). Los resultados de esta investigación serán publicados en reportes y revistas científicas de forma anónima. En ningún momento se mostrará información que permita la identificación de las personas que participan en esta encuesta. Los resultados representan, más bien, tendencias generales de la población.

Tenga en cuenta que nuestro objetivo no es evaluarlo ni persuadirlo; solamente queremos conocer su opinión. Usted podrá contestar libremente lo que piensa y representa mejor su punto de vista. También usted podrá retirarse del estudio si lo estima oportuno sin que ello le cause algún perjuicio alguno.

Si desea tener acceso a primeros resultados obtenidos en esta investigación, indíquelo, también, al final de la encuesta. Las direcciones de correo electrónico que se recolecten en esta investigación serán separadas de los datos antes de su análisis, se guardarán confidencialmente y no serán entregadas a terceros.

Si tiene preguntas o necesita más información, no dude en contactarnos vía correo electrónico a fredy.monge@unsaac.edu.pe.

¡Muchas gracias por su colaboración!

DECLARACIÓN Y/O CONSENTIMIENTO

Acepto voluntariamente participar en este estudio, comprendo de las actividades en las que participaré si decido ingresar al estudio, también entiendo que puedo decidir no participar y que puedo retirarme del estudio en cualquier momento.

Por favor, confirme:

*

Seleccione una de las siguientes opciones

Por favor seleccione sólo una de las siguientes opciones:

- He leído y entendido el texto anterior y acepto participar en la presente encuesta.
- No deseo (por el momento) participar en la presente encuesta.

INFORMACIÓN PERSONAL

AQUI SOLICITAMOS ALGUNOS DATOS DEL PARTICIPANTE

Sexo *

Por favor seleccione sólo una de las siguientes opciones:

- Femenino
- Masculino

Edad *

Por favor, escriba su respuesta aquí:

ANSIEDAD: GAD	No, en absoluto	Varios días	Más de la mitad de los días	Casi todos los días
	0	1	2	3
1 Me siento nervioso, ansioso o con los pelos de punta (al borde)				
2 No me siento capaz de detener o controlar la preocupación				
3 Me preocupo demasiado por cosas diferentes				
4 Tengo problemas o dificultades para relajarme				
5 Me siento muy inquieto y es difícil quedarme quieto				
6 Me molesto o irrito con facilidad				
7 Me siento asustado como si algo horrible pudiera pasar				

Amenazas percibidas al covid19

	Muy improbable	Improbable	ni improbable ni probable	Probable	Muy probable
	1	2	3	4	5
¿Cuán probable cree Usted que contraerá la nueva infección por coronavirus durante el próximo mes?					

	Muy leve	Leve	Moderado	Grave	Muy grave
	1	2	3	4	5
¿Qué tan grave cree que sería la nueva infección por coronavirus si la contrajera?					

	Mucho más baja	Más baja	Similar	Más alta	Mucho más alta
	1	2	3	4	5
¿La transmisibilidad del nuevo coronavirus (covid-19) en comparación con el SARS (de brote en el 2002) es?					
¿El daño al cuerpo del nuevo coronavirus (covid-19) en comparación con el SARS (de brote en el 2002) es?					

Grado de información al publico

	Muy en desacuerdo	Desacuerdo	Ni en desacuerdo ni de acuerdo	De acuerdo	muy de acuerdo
	1	2	3	4	5
¿La información que he recibido de parte del gobierno sobre el brote del nuevo coronavirus es suficiente?					

	Nunca	Casi nunca	A veces	Casi siempre	Siempre
	1	2	3	4	5

¿Con qué frecuencia se ha sentido confundido o preocupado sobre la confiabilidad de la información que recibían de parte del gobierno?						Reverse code

Confianza del publico

	Muy en desacuerdo	Desacuerdo	Ni en desacuerdo ni de acuerdo	De acuerdo	muy de acuerdo
	1	2	3	4	5
¿Creo que puedo tomar medidas para protegerme contra el coronavirus?					

Percepción de riesgo del covid-19

		Nada preocupado	2	3	4	5	6	Muy preocupado
1	¿Qué tan preocupado está usted personalmente por los siguientes problemas en la actualidad? - Coronavirus (COVID-19)							
		No es probable	2	3	4	5	6	Muy probable
2	¿Qué tan probable cree que sea que se verá afectado directa y personalmente por lo siguiente en los próximos 6 meses? - Contrayendo el coronavirus / COVID-19							
		No es probable	2	3	4	5	6	Muy probable
3	¿Qué tan probable cree que es que sus amigos y familiares en el país donde vive actualmente se verán directamente afectados por lo siguiente en los próximos 6 meses? - Contrayendo coronavirus / COVID-19							
		Totalmente en desacuerdo	2	3	4	Totalmente de acuerdo		
4	¿Cuánto está de acuerdo o en desacuerdo con las siguientes declaraciones? - El coronavirus / COVID-19, no afectará a muchas personas en el país en el que vivo actualmente.							

5	¿Cuánto está de acuerdo o en desacuerdo con las siguientes declaraciones? - Probablemente me enfermaré con el coronavirus / COVID-19.	Totalmente en desacuerdo	2	3	4	Totalmente de acuerdo
6	¿Cuánto está de acuerdo o en desacuerdo con las siguientes declaraciones? - Enfermarse con el coronavirus / COVID-19 puede ser grave	Totalmente en desacuerdo	2	3	4	Totalmente de acuerdo
