# Supplementary material for: Psychological Factors Affecting Risk Perception of COVID-19: Evidence from Peru and China
Source: Int J Environ Res Public Health. 2021 Jun 17;18(12):6513. doi: 10.3390/ijerph18126513 (PMC8296494; doi:10.3390/ijerph18126513)
Supplement: Supplementary file 1 [file ijerph-18-06513-s001.zip › S2.pdf]

Supplementary material S2

Comparisons of risk perception with age, anxiety, perceived threats, questions 1 and 2 of trust of information, self-confidence, sex and country.

Variable	Sample Total (*N* = 1594)	Low (*N* = 558)	Medium (*N* =776)	High (*N* = 260)	χ^2	*p*
	n(%)	*n*(%)	*n*(%)	*n*(%)		
Age					4.20	0.123
18 to 38 years	1340 (84)	472 (35.2)	661 (49.3)	207 (15.4)		
39 to 59 years	239 (15)	80 (33.5)	110 (46)	49 (20.5)		
60 years or more	14(1)	5 (35.7)	5 (35.7)	4 (28.6)		
GAD-7 (Anxiety)					109.53	0.001***
No anxiety	908 (57)	395 (43.5)	415 (45.7)	98 (10.8)		
Mild anxiety	474 (29.7)	114 (24.1)	259 (54.6)	101 (21.3)		
Moderate anxiety	158 (9.9)	39 (24.7)	79 (50)	40 (25.3)		
Severe anxiety	54 (3.4)	10 (18.5)	23 (42.6)	21 (38.9)		
Perceived threats to Covid-19					532.71	0.001***
Mild	50 (3)	33 (66)	10 (20)	7 (14)		
Moderate	832 (52.2)	411 (49.4)	355 (42.7)	66 (7.9)		
Serious	712 (44.7)	114 (16)	411 (57.7)	187 (26.3)		
Is the information I have received from the government about the outbreak of the new coronavirus sufficient? – Trust in Government Information1					69.82	0.001***
Strongly disagree	102 (6.4)	28 (27.5)	50 (49)	24 (23.5)		
Disagree	274 (17.2)	53 (19.3)	165 (60.2)	56 (20.4)		
Neither agree nor disagree	483 (30.3)	150 (31.1)	261 (54)	72 (14.9)		
Agree	586 (36.8)	241 (41.1)	257 (43.9)	88 (15)		
Strongly agree	149 (9.3)	86 (57.7)	43 (28.9)	20 (13.3)		
How often have you been confused or concerned about the reliability of the information you received from the government? - Trust in Government Information2					162.85	0.001***
Never	118 (7.4)	80 (67.8)	28 (23.7)	10 (8.5)		
Rarely	286 (17.9)	152 (53.1)	111 (38.8)	23 (8)		
Sometimes	828 (51.9)	255 (30.8)	440 (53.1)	133 (16.1)		
Usually	289 (18.1)	51 (17.6)	174 (60.2)	64 (22.1)		
Always	73 (4.6)	20 (27.4)	23 (31.5)	30 (41.1)		
Do I think I can take steps to protect myself against the coronavirus? – Self-confidence					5.42	0.247
Strongly disagree	47 (2.9)	17 (36.2)	16 (34)	14 (29.8)		
Disagree	63 (4)	17 (27)	36 (57.1)	10 (15.9)		

Neither agree nor disagree	184 (11.5)	66 (35.9)	100 (54.3)	18 (9.8)		
Agree	897 (56.3)	299 (33.3)	449 (50.1)	149 (16.6)		
Strongly agree	403 (25.3)	159 (39.5)	175 (43.4)	69 (17.1)		
					Wilcoxon	
Sex					12.84	0.001***
Female	821 (51.5)	229 (27.9)	435 (53)	157 (19.1)		
Male	773 (48.5)	329 (42.6)	341 (44.1)	103 (13.3)		
Country					13.03	0.001***
Perú	1092 (68.6)	185 (16.9)	675 (61.8)	232 (21.2)		
China	502 (31.4)	373 (74.3)	101 (20.1)	28 (5.6)		

Note: x^2: Kruskal Wallis and *W*: Wilcoxon, for nonparametric data
