# Supplementary material for: Psychological Factors Affecting Risk Perception of COVID-19: Evidence from Peru and China
Source: Int J Environ Res Public Health. 2021 Jun 17;18(12):6513. doi: 10.3390/ijerph18126513 (PMC8296494; doi:10.3390/ijerph18126513)
Supplement: Supplementary file 1 [file ijerph-18-06513-s001.zip › S3.pdf]

Supplementary material S3

Differences in the levels of the variables by country

	Total (%)	Country	
		Peru N (%)	China N (%)
Risk Perception			
Low	558 (35%)	185 (33.2%)	373 (66.8%)
Medium	776 (48.7%)	675 (87%)	101 (13%)
High	260 (16.3%)	232 (89.2%)	28 (10.8%)
Total	1594 (100%)	1092 (68.5%)	502 (31.5%)
Perceived threats to Covid-19			
Mild	50 (3.1%)	25 (50%)	25(50%)
Moderate	832 (52.2%)	458(55%)	374 (45%)
Serious	712 (44.7%)	609 (85.5%)	103 (14.5%)
Total	1594 (100%)	1092 (68.5%)	502 (31.5%)
Anxiety			
No Anxiety	908 (57%)	591 (65.1%)	317(34.9%)
Mild anxiety	474(29.7%)	352 (74.3%)	122(25.7%)
Moderate Anxiety	158(9.9%)	114 (72.2%)	44 (27.8%)
Severe Anxiety	54(3.4%)	35 (64.8%)	19 (35.2%)
Total	1594 (100%)	1092 (68.5%)	502 (31.5%)
Is the information I have received from the government about the outbreak of the new coronavirus sufficient? – Trust in Government Information1			
Strongly disagree	102 (6.4%)	85(83.3%)	17(16.7%)
Disagree	274 (17.2%)	261(95.3%)	13(4.7%)
Neither agree nor disagree	483(30.3%)	351(72.7%)	132(27.3%)
Agree	586(36.8%)	348(59.4%)	238(40.6%)
Strongly agree	149(9.3%)	47(31.5%)	102(68.5%)
Total	1594(100%)	1092(68.5%)	502(31.5%)
How often have you been confused or concerned about the reliability of the information you received from the government? - Trust in Government Information2			
Never	118(7.4%)	41(34.7%)	77(65.3%)
Rarely	286(17.9%)	142(49.7%)	144(50.3%)
Sometimes	828(51.9%)	617(74.5%)	211(25.5%)
Usually	289(18.1%)	239(82.7%)	50(17.3%)
Always	73(4.6%)	53(72.6%)	20(27.4%)
Total	1594(100%)	1092(68.5%)	502(31.5%)
Do I think I can take steps to protect myself against the coronavirus? – Self-confidence			
Strongly disagree	47 (2.9%)	32(68.1%)	15 (31.9%)
Disagree	63 (4%)	50 (79.4%)	13 (20.6%)
Neither agree nor disagree	184 (11.5%)	113 (61.4%)	71 (38.6%)
Agree	897 (56.3%)	635 (70.8%)	262 (29.2%)
Strongly agree	403 (25.3%)	262 (65%)	141 (35%)
Total	1594 (100%)	1092 (68.5%)	502 (31.5%)
